# Possible Protective Effects of TA on the Cancerous Effect of Mesotrione

**DOI:** 10.3390/nu12051343

**Published:** 2020-05-08

**Authors:** Agata Jabłońska-Trypuć, Urszula Wydro, Elżbieta Wołejko, Joanna Rodziewicz, Andrzej Butarewicz

**Affiliations:** 1 Division of Chemistry, Biology and Biotechnology, Faculty of Civil Engineering and Environmental Sciences, Bialystok University of Technology, 15-351 Białystok, Poland; u.wydro@pb.edu.pl (U.W.); e.wolejko@pb.edu.pl (E.W.); a.butarewicz@pb.edu.pl (A.B.); 2 Faculty of Geoengineering, Department of Environmental Engineering, University of Warmia and Mazury in Olsztyn, 10-719 Olsztyn, Poland; joanna.rodziewicz@uwm.edu.pl

**Keywords:** mesotrione, traumatic acid, breast cancer, herbicide, antioxidant, oxidative stress

## Abstract

The interaction of different food ingredients is now a very important and often emerging topic of research. Pesticides and their breakdown products, which may be carcinogenic, are one of the frequently occurring food contaminants. Compounds like traumatic acid (TA), which originates from plants, are beneficial, antioxidant, and anticancer food ingredients. Previously obtained results from our research group indicated antioxidative in normal human fibroblasts and prooxidative in cancer cells activity of TA. Since the literature data show an undoubted connection between the presence of pesticides in food and the increased incidence of different types of cancers, we attempted to clarify whether TA can abolish the effect of mesotrione stimulating the growth of cancer cells. In order to study the influence of mesotrione on breast cancer cells, we decided to carry out cytotoxicity studies of environmentally significant herbicide concentrations. We also analyzed the cytotoxicity of TA and mixtures of these two compounds. After selecting the most effective concentrations of both components tested, we conducted analyses of oxidative stress parameters and apoptosis in ZR-75-1 cells. The obtained results allow us to conclude that traumatic acid by stimulating oxidative stress and apoptosis contributes to inhibiting the growth and development of cells of the ZR-75-1 line strengthened by mesotrione. This may mean that TA is a compound with pro-oxidative and proapoptotic effects in cancer cells whose development and proliferation are stimulated by the presence of mesotrione. The presented results may be helpful in answering the question of whether herbicides and their residues in edibles may constitute potential threat for people diagnosed with cancer and whether compounds with proven pro-oxidative effects on cancer cells can have potential cytoprotective functions.

## 1. Introduction

Different chemical substances from the group of pesticides are used in the food production process to ensure seasonal availability and good quality of products. However, it should be mentioned that the frequent and widespread use of pesticides carries the risk of their penetration into the human body. Pesticides are a group of chemical compounds, both natural and synthetic, that are used in order to destroy plant and animal parasites, reduce the risk of plant diseases, and control weeds. The massive use of pesticides results from the growing number of consumers, and thus from an increased demand for food. Pesticide residues may remain in food after their application to crops. The maximum permissible levels of pesticides residues in food are determined by the regulatory authorities in the European Union, mostly at the level of the European Commission. Exposure of a given population to pesticides and their residues most often occurs as a result of the consumption of processed food or close contact with pesticide treated areas, such as farms [1,2]. 

Mesotrione (Mes) is an herbicide which controls most broadleaf weeds and weed grasses in crops cultivation. Due to its frequent use in agriculture, the detectable level of this compound in countries such as North America and Canada fluctuates around 4.1µg/L [3,4]. According to its chemical structure, Mes is 2-[4-methylsulfonyl-2-nitrobenzoyl]-1,3-cyclohexanedione and it belongs to the family of triketone. Its main role is an inhibition of the enzyme 4-hydroxy-phenyl-pyruvate-dioxygenase, which converts tyrosine to plastoquinone (PQ) and alpha-tocopherol [5]. Mes is a quite water soluble compound, which, in combination with its widespread application and soil retention capacity, contributes to the fact that this herbicide easily contaminates surface and groundwater [6]. According to Bonnet et al., Mes decomposition products appear to be more dangerous and harmful than the parent compound [7].

The endocrine-disrupting influence of selected pesticides has caused great concerns due to the hormonal activity of many well-documented risk factors for breast cancer. Literature data suggest that selected pesticides could be related to an increase in breast cancer risk and urge researchers to examine environmental risk factors and possible compounds, preferably of natural origin, that could reduce side effects of pesticide use and thus the incidence of this disease [8,9,10]. Many pesticides are considered as analogues of human hormones. They may exert influence through estrogen receptors. Therefore, we chose to examine the ZR-75-1 breast cancer cell line, which is commonly used for endocrine-based research, rather than choosing on the other breast cancer cell line or nonhuman cell line. Discordances in scientific data regarding possible herbicides cancerogenic properties may result from the different test models. Therefore, for the experiment, we decided to choose the human estrogen-dependent breast cancer cell line, which is characterized by the presence of the estrogen receptor (ER+). The aim of this paper was to study the mutual interaction mechanisms of two opposite compounds, one of which is highly undesirable food contaminant (Mes), and traumatic acid (TA), which is a promising food ingredient. In already published papers, we indicated that TA is characterized by antioxidative activity in normal human fibroblasts and pro-oxidative properties in malignant cells [11,12]. In our preliminary studies on the toxicological effects of Mes and TA in various breast cancer lines, we showed that TA, depending on the concentration used, has an effect on the selected herbicides including Mes [13]. The literature, which has documented relationship between the consumption of food contaminated with pesticides and the increased incidence of different types of cancers, led us to investigate whether TA can counteract the stimulating influence of mesotrione on the proliferation and growth of malignant cells.

## 2. Materials and Methods

### 2.1. Reagents

Phosphate buffered saline (PBS), without Ca and Mg, was provided PAN Biotech (Aidenbach, Germany). SDS (Sodium dodecyl sulphate), TCA (trichloroacetic acid), TBA (thiobarbituric acid), Folin-Ciocalteu reagent, and Mesotrione were provided by Sigma-Aldrich and DTNB (dithiobis-2-nitrobenzoic acid, Ellman’s reagent) by Serva. Dichlorodihydrofluorescein diacetate assay (DCFH-DA) and cell stain double staining kit containing propionium iodide and calcein AM was provided by Sigma-Aldrich, St. Louis, MO, USA. The fluorescein isothiocyanate (FITC) Annexin V Apoptosis Detection Kit I was purchased from BD Pharmingen (San Diego, CA, USA). The Cayman’s Catalase Assay Kit, Cayman’s Superoxide Dismutase Assay Kit, and Cayman GPx Assay were obtained from Cayman Chemical (Ann Arbor, MI, USA).

### 2.2. Cell Culture

The effect of herbicide, TA (Cayman Chemical Company (1180 East Ellsworth Road, Ann Arbor, MI, USA), purity ≥ 98%; formal name: 2E-dodecenedioic acid; CAS number: 6402-36-4; formulation: A crystalline solid) and the mix of herbicide with TA was studied in the ZR-75-1 cell line, which was obtained from American Type Culture Collection (ATCC, Manassas, VA, USA). The ZR-75-1 cells were cultured in RPMI-1640 Medium containing glucose at 4.5 mg/mL (25 mM) supplemented with 10% fetal bovine serum (FBS) (PAN Biotech), penicillin (100 U/mL) (PAN Biotech), and streptomycin (100 μg/mL) (PAN Biotech) at 37 °C in a humidified atmosphere of 5% CO_2_ in air.

### 2.3. Cytotoxicity Assay 

Cytotoxicity were studied according to the method of Carmichael using 3-(4,5-dimethylthiazol-2-yl)-2,5-diphenyltetrazolium bromide (MTT) (Sigma-Aldrich, St. Louis, MO, USA) [14].

TA cytotoxicity was studied at selected concentrations of 0.5 µM, 0.75 µM, 1 µM, 10 µM, 20 µM, 50 µM, 100 µM, 200 µM, 500 µM, 750 µM, and 1000 µM. Mes cytotoxicity was estimated at concentrations of 0.01 µM, 0.025 µM, 0.05 µM, 0.1 µM, 0.5 µM, 1 µM, 5 µM, 10 µM, 25 µM, and 50 µM. The concentrations of both compounds selected for the analysis of the effect of the tested chemicals mixture on the cells have been chosen on the basis of MTT cytotoxicity tests performed. The concentration of Mes with the highest stimulating effect on the ZR-75-1 cells was selected. The control cells were cultured without test compounds. 

Breast cancer cells were seeded in a 96-well plate at a density of 2 × 10^4^ cells/well. Cells cultured for 24 h and 48 h were first treated with TA in the concentration range from 0.5 µM to 1000 µM, then Mes in the concentration range from 0.01 µM to 50 µM, and finally TA mixed with Mes: TA in the concentration range from 0.5 µM to 1000 µM mixed with 0.05 µM Mes. The analysis was conducted according to Jabłońska-Trypuć et al. using a microplate reader GloMax^®^-Multi Microplate Multimode Reader (Promega Corporation, Madison, WI, USA) [13]. The viability of breast cancer cells was presented as a percentage of control cells. All the experiments were done in triplicate.

### 2.4. Caspase 3/7 Activity Assay

The activity of caspases 3/7 was examined at TA concentrations of 100µM and 200 µM, Mes concentration of 0.05 µM, and the combination of two compounds (TA + Mes, concentrations: 100 µM + 0.05 µM and 200 µM + 0.05 µM, respectively) after 24 h and 48 h of incubation. Breast cancer cells were seeded in 96-well white plate at a density of 2 × 10^4^ cells/well. Luminescent assay was applied according to manufacturer’s instructions (Promega Corporation, Madison, WI, USA) as described previously [15]. A microplate reader GloMax^®^-Multi Microplate Multimode Reader was used and the experiments were done in triplicate.

### 2.5. Fluorescent Microscopy Analysis

For the analysis of apoptotic and necrotic cells nuclear morphology, two fluorescent dyes, propionium iodide and calcein-AM, were applied. Cells were seeded on cell imaging dishes with coverglass bottoms at a density of 2 × 10^5^ cells/well with 200 µM TA, 0.05 µM Mes, the mix of two compounds, and without the tested compound for 24 h. Subsequently, cells were washed twice with PBS and then stained with dyes solution in the dark in 37 °C for 15 min. The mixture of dyes was removed and the cells were washed with phosphate buffer and analyzed with the use of Olympus IX83 fluorescent microscope with SC180 camera with Cell Sens Dimension 1.17 program (200 × magnification). Calcein-AM stains viable cells and PI pass only through damaged membrane in dead cells. The following criteria were used: Living cells were characterized by regularly distributed green chromatin nucleus and are stained with green color. Dead cells, probably apoptotic cells, were characterized by red nuclei with chromatin condensation or fragmentation. Necrotic cells showed red-stained cell nuclei.

### 2.6. Analysis of Apoptosis Using Flow Cytometry

Breast cancer cells were seeded in six-well plates at a density of 2 × 10^5^ cells/well. Cells were exposed to 100 µM TA, 200 µM TA, 0.05 µM Mes, and the mix of two compounds (TA + Mes, concentrations: 100 µM + 0.05 µM, 200 µM + 0.05 µM, respectively) and incubated for 24 h and 48 h. Apoptosis was studied by flow cytometry on FACSCalibura II cytometer (Becton-Dickinson). After trypsinization, cells were resuspended in RPMI-1640. Then, cells were suspended in binding buffer for staining with FITC (Annexin V) and propidium iodide (PI) for 15 min at room temperature in the dark following the manufacturer’s instructions (FITC Annexin V apoptosis detection Kit I). The signal obtained from cells stained with Annexin V or PI alone was used for fluorescence compensation. Data were analyzed with FACStationTM software (BD PharmingenTM, San Diego, CA, USA).

### 2.7. Total Protein Content

ZR-75-1 cells were exposed to 100 µM TA, 200 µM TA, 0.05 µM Mes, and the mix of two compounds (TA + Mes, concentrations: 100 µM + 0.05 µM, 200 µM + 0.05 µM, respectively) and incubated for 24 h and 48 h. ZR-75-1 cells (2.5 × 10^5^ cells/mL) were cultured with tested compounds. The protein concentration was determined as described previously [15]. All the experiments were done in triplicate.

### 2.8. Determination of SH Groups

SH groups content was analyzed using Rice-Evans method (1991) as described previously [15]. ZR-75-1 cells were exposed to 100 µM TA, 200 µM TA, 0.05 µM Mes, and the mix of two compounds (TA + Mes, concentrations: 100 µM + 0.05 µM, 200 µM + 0.05 µM, respectively) and incubated for 24 h and 48 h. ZR-75-1 cells (2.5 × 10^5^ cells/mL) were cultured with tested compounds. All the experiments were done in triplicate.

### 2.9. Determination of TBA Reactive Species (TBARS) Level

The Rice-Evans method (1991) was used for measuring membrane lipid-peroxidation products level (TBARS), as described previously [15]. ZR-75-1 cells were exposed to 100 µM TA, 200 µM TA, 0.05 µM Mes, and the mix of two compounds (TA + Mes, concentrations: 100 µM + 0.05 µM, 200 µM + 0.05 µM, respectively) and incubated for 24 h and 48 h. ZR-75-1 cells (2.5 × 10^5^ cells/mL) were cultured with test compounds. All the experiments were done in triplicate.

### 2.10. Determination of GSH/GSSG

GSH/GSSG (GSH–reduced form of glutathione, GSSG–oxidized form of glutathione) ratio was examined at TA concentrations of 100 µM and 200 µM, Mes concentration of 0.05 µM, and the mixture of these two compounds (TA + Mes, concentrations: 100 µM + 0.05 µM and 200 µM + 0.05 µM, respectively) after 24 h and 48 h of incubation. Breast cancer cells were seeded in 96-well white plates at a density of 2 × 10^4^ cells/well. GSH/GSSG ratio was assayed in triplicate via GSH/GSSG-Glo™ kit (Promega Madison, WI, USA) following manufacturer’s instructions as described [15]. 

### 2.11. Intracellular ROS Detection

Intracellular ROS level was examined at TA concentrations of 100 µM and 200 µM, Mes concentration of 0.05 µM, and the mixture of these two compounds (TA + Mes, concentrations: 100 µM + 0.05 µM and 200 µM + 0.05 µM, respectively) after 24 h and 48 h of incubation. Breast cancer cells were seeded in 96-well white plates at a density of 2 × 10^4^ cells/well. Dichlorodihydrofluorescein diacetate (DCFH-DA), (Sigma, St. Louis, MO, USA) and GloMax^®^-Multi Detection System (Promega Corporation, Madison, WI, USA) were used in order to measure the level of intracellular reactive oxygen species (ROS) [16]. The method was described previously [12]. All the experiments were done in triplicate.

### 2.12. Catalase Activity

Catalase is an enzyme which is involved in the detoxification processes, mainly the metabolism of hydrogen peroxide, which originates during normal aerobic metabolism and pathogenic reactive oxygen species (ROS) generation. Cells were cultured in six-well plates at 1 × 10^5^ cells/well (Sarstedt), treated with 100 µM and 200 µM TA, 0.05 µM Mes, and the mix of two compounds (TA + Mes, concentrations: 100 µM + 0.05 µM, 200 µM + 0.05 µM, respectively) for 24 h and 48 h. For the determination of catalase activity, the Catalase Assay Kit (Cayman Chemical Company Ann Arbor, MI, USA) was used following manufacturer’s instructions. The absorbance of final product was read at 540 nm using the GloMax^®^-Multi Microplate Multimode Reader. All the experiments were done in triplicate. 

### 2.13. Glutathione Peroxidase Activity

Glutathione peroxidase is involved in cells protection against oxidative stress by catalyzing the reduction of hydroperoxides by reduced GSH. Cells were cultured in six-well plates at 1 × 10^5^ cells/well (Sarstedt), and treated with 100 µM and 200 µM TA, 0.05 µM Mes, and the mix of two compounds (TA + Mes, concentrations: 100 µM + 0.05 µM, 200 µM + 0.05 µM, respectively) for 24 h and 48 h. Prior to the analysis, cells were washed with phosphate buffer. Cells were collected using rubber policeman and homogenized in a cold buffer, then centrifuged at 10,000× *g* for 15 min at 4 °C. Supernatant was used for the assay. For the determination of glutathione peroxidase activity GPx Assay kit (Cayman Chemical Company Ann Arbor, MI, USA) was used following manufacturer’s instructions. The absorbance at 340 nm was read using the GloMax^®^-Multi Microplate Multimode Reader. All the experiments were done in triplicate.

### 2.14. Superoxide Dismutase Activity

Superoxide dismutases belong to the group of metalloenzymes. They catalyze the superoxide anion dismutation to molecular oxygen and hydrogen peroxide and therefore play an important role in the cellular antioxidant defense system. Cells were seeded in six-well plates at 1 × 10^5^ cells/well (Sarstedt), and treated with 100 µM and 200 µM TA, 0.05 µM Mes, and the mix of two compounds (TA + Mes, concentrations: 100 µM + 0.05 µM, 200 µM + 0.05 µM, respectively) for 24 h and 48 h. Prior to the analysis, cells were washed with phosphate buffer. Cells were collected using rubber policeman and homogenized in a cold buffer, then centrifuged at 10,000× *g* for 15 min at 4 °C. Supernatant was used for the assay. For the determination of SOD activity, the Superoxide Dismutase Assay Kit (Cayman Chemical Company Ann Arbor, MI, USA) was applied following manufacturer’s instructions. The absorbance (440–460 nm) was read using the GloMax^®^-Multi Microplate Multimode Reader. All the experiments were done in triplicate.

### 2.15. Statistical Analysis

Statistical analysis for the obtained results was performed. The effect of TA, Mes, and the combination of TA and Mes on apoptosis and oxidative stress parameters in ZR-75-1 cells was calculated as a means and compared in analysis of variance using the post-hoc test of ANOVA. The significant differences were estimated by Tukey test at *p* < 0.05. A biplot graph was used in order to present correlation between parameters. Staistica 13 (StatSoft, Kraków, Poland) was used to present data analysis.

## 3. Results 

### 3.1. Cytotoxicity

**Figure 1:**
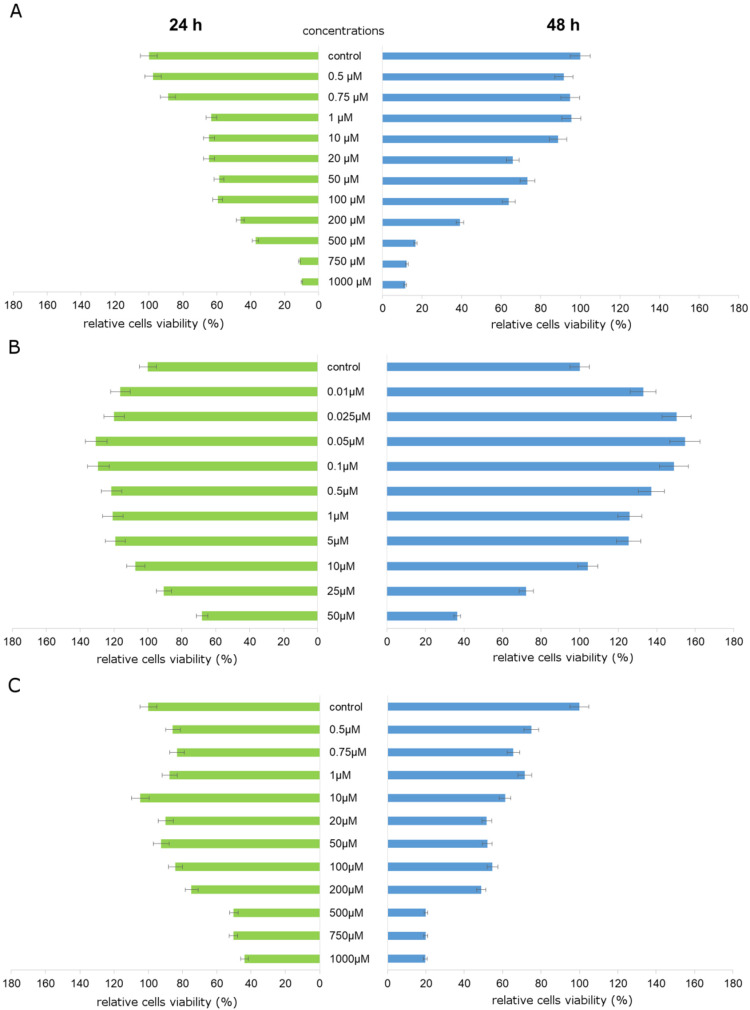
Relative cell viability. The ZR-75-1 cell line was exposed to (**A**) graded concentrations of TA (traumatic acid), (**B**) graded concentrations of Mes (mesotrione), and (**C**) graded concentrations of TA mixed with 0.05 µM of Mes. Mean values from three independent experiments ± standard deviation (SD) are shown.

An MTT assay was applied in order to estimate potential TA cytotoxicity (Figure 1A). The analyzed compound caused significant decreases in relative ZR-75-1 cell viability, which was observed right after 24 h treatment. It was effective even in lower concentrations. At 1 µM TA concentration, a 37% decrease was noticed after 24 h treatment. Concentrations of 100 µM and 200 µM decreased the viability of cells by about 40% and more than 50%, respectively, after 24 h treatment. None of the tested TA concentrations stimulated studied cells viability. On the other hand, Mes significantly increased relative cell viability. The most significant increase in the relatively shorter time of incubation was observed under the influence of 0.05 µM of Mes, which is presented in Figure 1B. Therefore, one of Mes concentrations, 0.05 µM, was selected for further analysis, and was applied together with all of the studied concentrations of TA. We noticed significant decline in relative cell viability in both incubation times, especially in combination of Mes with TA in 0.05 µM + 100 µM and 0.05 µM + 200 µM, respectively (Figure 1C). Taking into account the results of the above-mentioned experiments, we decided to choose two combinations of analyzed compounds for further analysis of the mechanisms by which they affect breast cancer cells. TA concentrations of 100 µM and 200 µM are close to IC50 value, and more importantly, TA concentrations higher than 400 µM could be potentially cytotoxic for the human organism. For studying oxidative stress parameters and apoptosis, we analyzed the influence of 0. 05 µM Mes + 100 µM TA and 0.05 µM Mes + 200 µM TA. 

### 3.2. Apoptosis

**Figure 2:**
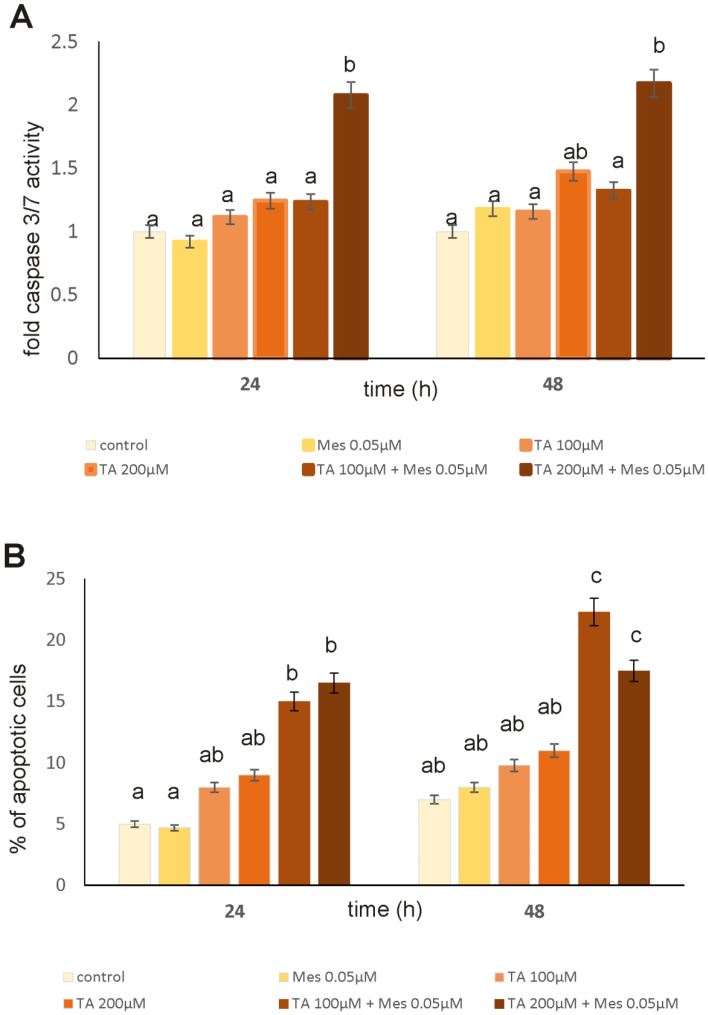
The effect of TA, Mes, and the combination of TA+Mes on apoptosis in ZR-75-1 cells, which were incubated with 100 µM of TA, 200 µM of TA, 0.05 µM of Mes, and a mix of 100 µTA + 0.05 µM Mes, 200 µM TA + 0.05 µM Mes. (**A**) Caspase 3/7 activity in ZR-75-1 cells under the influence of TA, Mes and TA + Mes on, (**B**) Bar graphs presenting the percentage of apoptotic cells. Mean values from three independent experiments ± SD are shown. Different letters indicate statistical differences (*p* ≤ 0.05) between treatments estimated by Tukey’s test.

Before conducting the Caspase-Glo^®^ 3/7 Assay, cells were subjected to 0.05 µM Mes, 100 µM TA, 200 µM TA, and the combination of 0.05 µM of Mes with 100 µM TA and 0.05 µM of Mes with 200 µM of TA (Figure 2A). Mes treatment did not induce apoptosis in studied cell line. TA slightly enhanced the activity of analyzed caspases, especially after 48 h of treatment. However, the most significant changes in caspases 3/7 activity caused by two mixed compounds were observed when TA concentration was about 200 µM.

Apoptosis was also estimated using flow cytometry, and the results are presented in Figure 2B. In Figure 2B, the percent of apoptotic cells cultured for 24 h and 48 h with TA, Mes, and the mixture of TA and Mes is depicted. The values obtained for herbicide treatment indicate that tested compound did not enhance apoptosis. However, TA treatment, both alone and in combination with Mes, enhanced apoptosis. The results obtained in the flow cytometry experiment confirmed the studied caspases activity. 

**Figure 3:**
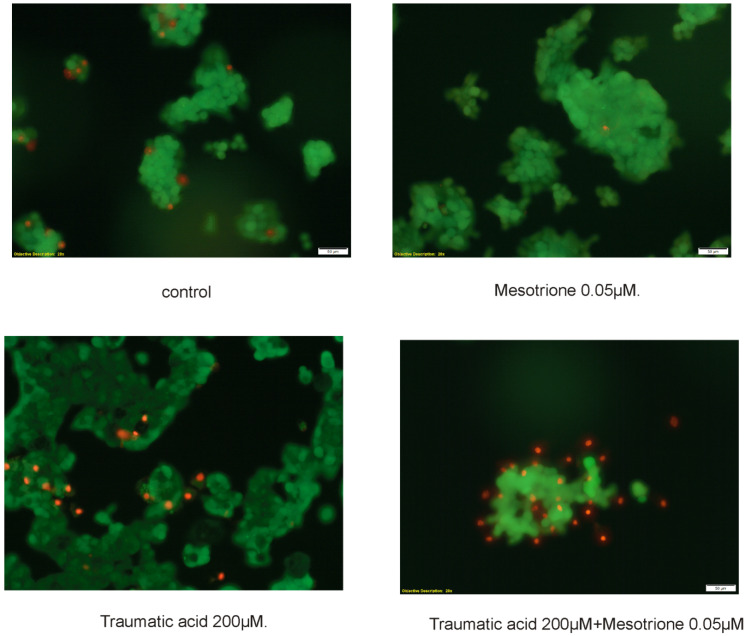
The influence of TA (200 µM), Mes (0.05 µM), and the mix of TA + Mes (200 µM + 0.05 µM) on apoptosis and necrosis in the ZR-75-1 cell line estimated using fluorescence microscope assay (200 × magnification). The cells were cultured with TA and Mes for 24 h and stained with Calcein-AM and propidium iodide. Three independent experiments were conducted and representative images are depicted.

A fluorescent microscopy assay was used in order to confirm the occurrence of apoptosis (Figure 3). We evaluated apoptotic and necrotic cells morphology using fluorescent staining. Similar to luminescence and flow cytometry analysis, we observed differences between control, TA, and TA + Mes treatments. We did not observe differences between control and pesticide-treated cells. Calcein—AM stained only viable cells, while propidium iodide stained viable and death cells.

### 3.3. Oxidative Stress

In the combination of Mes and TA, unsaturated dicarboxylic fatty acid demonstrates anticancer properties against Mes-induced breast cancer development by enhancing the stimulatory effect on oxidative stress parameters.

**Figure 4:**
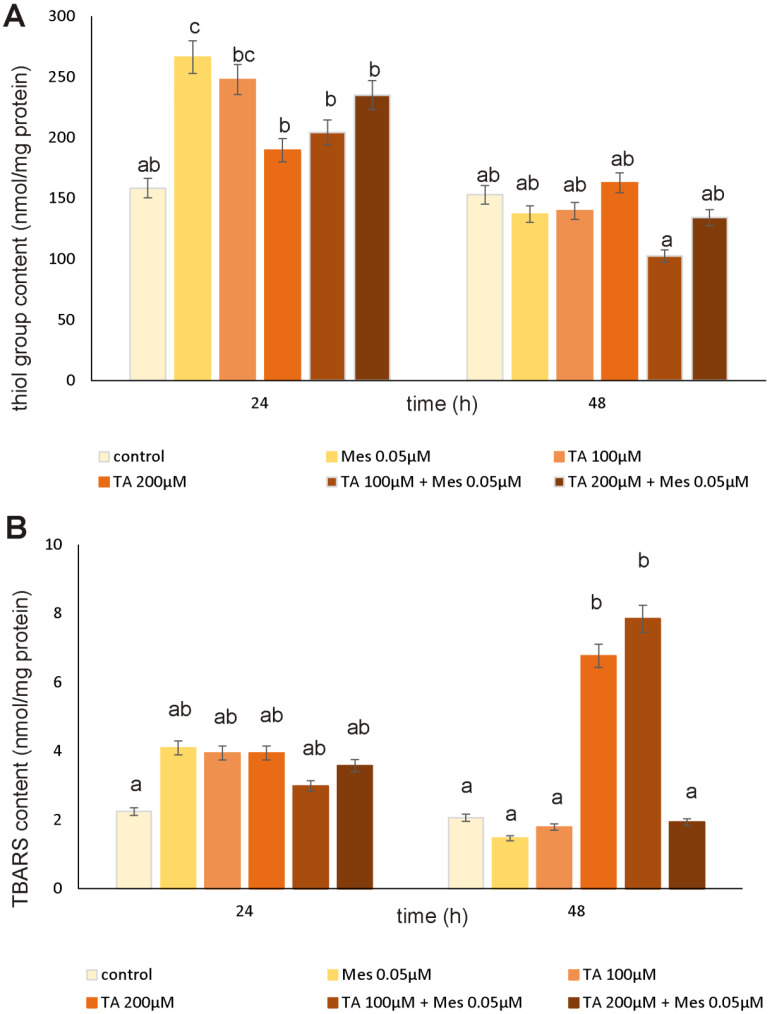
The influence of TA, Mes, and the mix of TA and Mes on SH group content (**A**) and TBARS content (**B**) in ZR-75-1 cells. The cells were cultured with 100 µM of TA, 200 µM of TA, 0.05 µM of Mes, mix of 100 µM TA + 0.05 µM Mes, and 200 µM TA + 0.05 µM Mes for 24 h and 48 h. Mean values from three independent experiments ± SD are shown. Different letters indicate statistical differences (*p* ≤ 0.05) between each treatment estimated by Tukey’s test.

The influence of TA and Mes on the amount of SH groups is presented in Figure 4A. A statistically significant increase in thiol group content by approximately 68% was noticed under the influence of Mes after 24 h. Exposure to TA after 24 h incubation also caused an increase in analyzed parameter. However, 48 h incubation with tested compounds caused a significant decrease in thiol group content. Notably, the decrease was observed in case of the TA pretreatment of cells. The obtained results indicate that 100 µM of TA delayed the antioxidative effect of Mes on breast cancer cells, because we noticed a decrease in thiol group content caused by 0.05 µM Mes in the culture pretreated with 100 µM TA. The presented data may indicate that TA could be a compound, which intensifies oxidative stress in cancer cells, even in the presence of herbicide.

Lipid peroxidation in cancer cells is a very important process, which consists a source of free radicals inevitable for fast cancer cell proliferation (Figure 4B). Incubation with all of the analyzed compounds after 24 h caused an increase in TBARS content, which was analyzed as an index of lipid peroxidation. However, statistically significant changes in tested parameter were observed after 48 h treatment. Both TA alone and 100 µM TA in combination with Mes caused a very high increase in TBARS level. However, 200 µM TA mixed with Mes caused a decrease of about 75% as compared to the first analyzed mix, which could be explained by the presence of the other lipid peroxidation products, for example HNE. The obtained results suggest that TA may demonstrate protective properties by increasing membrane phospholipid peroxidation to such a high level, which is toxic to cancer cells. 

**Figure 5:**
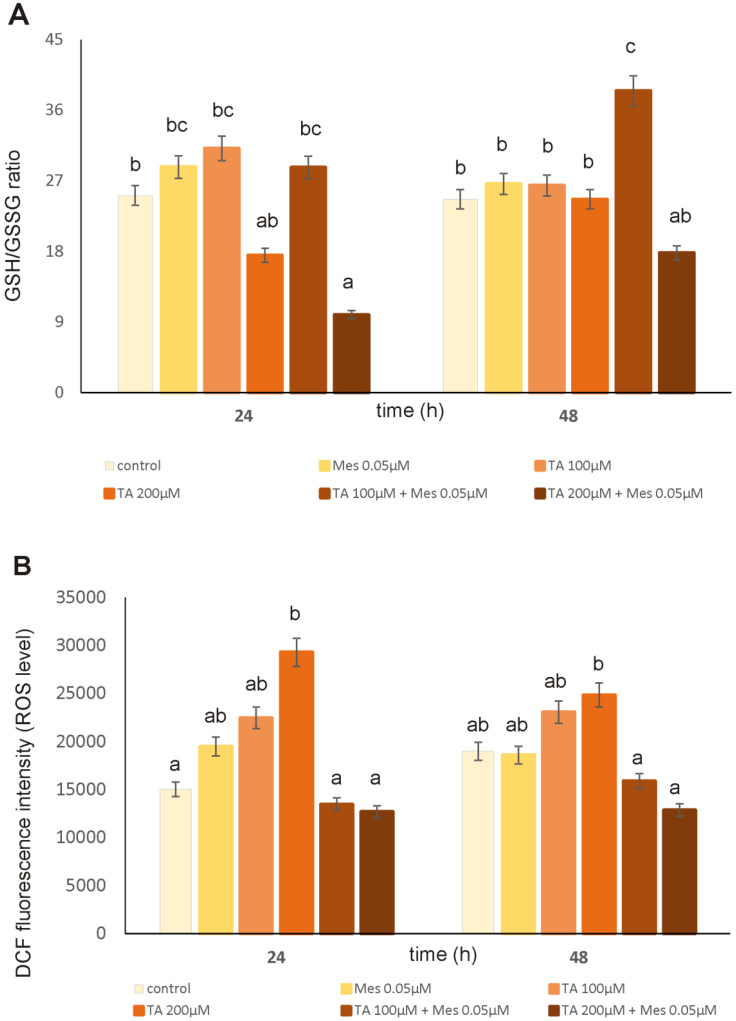
The influence of TA, Mes and the mix of TA and Mes on GSH/GSS (GSH–reduced form of glutathione, GSSG–oxidized form of glutathione) ratio (**A**) and reactive oxygen species (ROS) content (**B**) in ZR-75-1 cells. The cells were cultured with 100 µM TA, 200 µM TA, 0.05 µM Mes, and a mix of 100 µTA + 0.05 µM Mes, 200 µM TA + 0.05 µM Mes for 24 h and 48 h. Mean values from three independent experiments ± SD are shown. Different letters indicate statistical differences (*p* ≤ 0.05) between each treatment estimated by Tukey’s test.

Figure 5B shows the influence of TA and Mes on the production of ROS in the ZR-75-1 breast cancer cells. The intensity of fluorescence of 2′7′-dichlorodihydrofluorescein (DCF) for the ZR-75-1 cells cultured with TA and Mes for 24 h and 48 h is shown as a relative ROS amount. An incubation of cells with tested compounds caused an increase in ROS content in both analyzed times. At a concentration of 200 µM, TA caused an increase, which was very high, at about 95% after 24 h. Treatment with 100 µM and 200 µM of TA significantly reduced the ROS amount in the Mes-treated cell culture as compared to TA-treated cells. The presented data show an enhancing effect of TA on ROS formation.

The influence of Mes, TA, and the mixture of Mes with TA on GSH/GSSG ratio is depicted in Figure 5A. Reduced glutathione belongs to the group of very important antioxidants, which maintain oxidative balance within the cell. At a concentration of 200 µM, TA significantly decreased GSH/GSSG ratio after 24 h of incubation, while 100 µM of TA combined with 0.05 µM Mes significantly increased the tested parameter after 48 h incubation time compared to the control. Treatment with 200 µM of TA caused a reduced ratio of GSH/GSSG after 24 h and 48 h of culturing, even after addition of Mes. Treatment with the Mes and TA mixture for 24 h reduced the level of GSH compared to untreated cells. Based on the results obtained, we conclude that TA had a rather inhibitory effect and Mes had a stimulatory effect on the GSH/GSSG ratio in the ZR-75-1 cell line.

**Figure 6:**
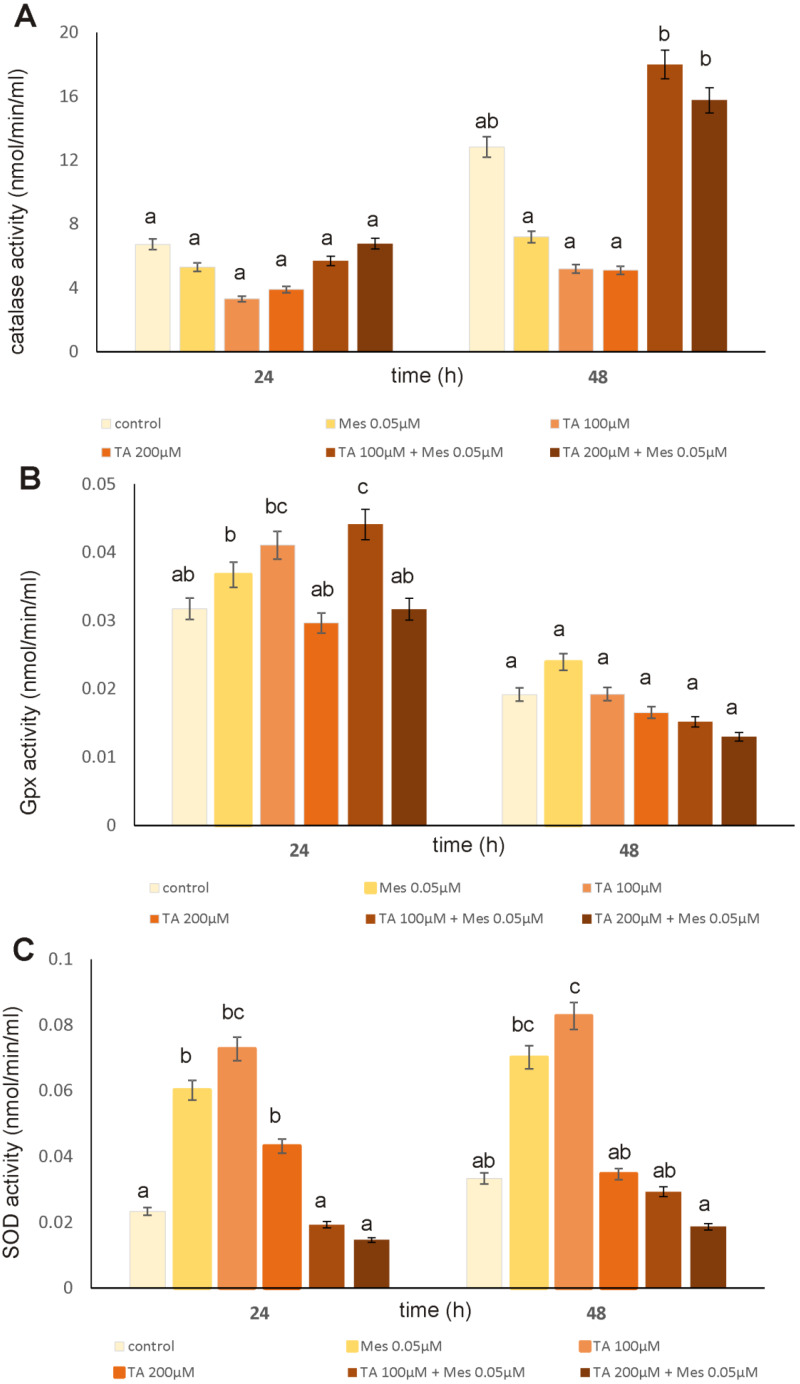
The influence of TA, Mes, and the mix of TA and Mes on catalase activity (**A**) GPx (glutathione peroxidase) activity (**B**) and SOD (superoxide dismutase) activity (**C**) in ZR-75-1 cells. The cells were cultured with 100 µM of TA, 200 µM of TA, 0.05 µM of Mes, and a mix of 100 µM TA + 0.05 µM Mes, 200 µM TA + 0.05 µM Mes for 24 h and 48 h. Mean values from three independent experiments ± SD are shown. Different letters indicate statistical differences (*p* ≤ 0.05) between each treatment estimated by Tukey’s test.

The first line of antioxidant defense that plays a key role in maintaining redox homeostasis in the cell are enzymes such as GPx, catalase and SOD. A significant increase in catalase activity under the influence of TA combined with 0.05 µM Mes was observed after 48 h incubation only (Figure 6A). At concentrations of 100 µM and 200 µM, TA, applied as a pretreatment before adding Mes, increased catalase activity by about 40% and 23%, respectively, as compared to the control untreated cells. At a concentration of 0.05 µM, Mes decreased catalase activity in both treatment times, however statistically insignificantly. The opposite results were observed in case of glutathione peroxidase (Figure 6B). The GPx activity was the highest under the influence of 100 µM TA + 0.05 µM Mes after 24 h incubation. Longer treatment with all of the tested compounds caused declines in GPx activity, but statistically insignificant. Our results showed that SOD activity was enhanced not only by the action of Mes, but also TA. The combination of these two compounds revealed decreases in SOD activity as compared to free Mes or free TA (Figure 6C).

**Figure 7:**
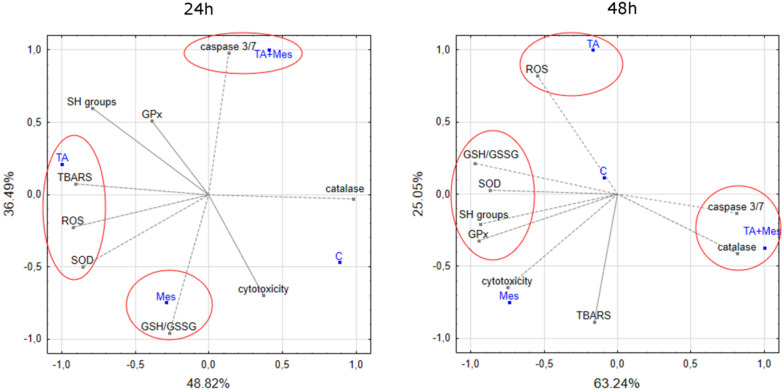
Biplot showing variables (SH groups (thiol groups), TBARS (thiobarbituric acid reactive species) content, GSH/GSSG ratio, ROS content, SOD, catalase, GPX activity, caspase 3/7 activity, cytotoxicity) and cases (tested compounds: Mes, TA, and TA+Mes—mix of two analyzed compounds) in two dimensions.

In Figure 7, PCA analysis is depicted. It presents the correlation between the studied variables concerning the oxidative stress and apoptosis resulting from the activity of tested compounds and their mixture in the ZR-75-1 cell line. Figure 7 shows that 24 h treatment with TA was positively correlated with TBARS content, ROS content, and SOD activity, and was also correlated with the first component, which explains the 48.82% variability. However, Mes treatment was correlated with GSH/GSSG ratio, which is represented by the second component, explaining the 36.49% variability. After 48 h treatment, the previously observed correlation between TA and ROS content and TA + M and caspase 3/7 activity was maintained.

## 4. Discussion

Due to the increasing popularity of diets based mainly on products of plant origin, we should be aware of the presence in everyday meals of both ingredients having a beneficial effect on the human body, but also compounds that are residues from the method of growing and preserving food of plant origin. The first group of compounds mentioned above includes TA, which is a plant hormone with beneficial antioxidant and probably anticancer effects. While the second group of compounds present in the crop plants are undoubtedly pesticides, an example of which is analyzed Mes. Taking into account its chemical structure TA belongs to the group of unsaturated fatty acids, whose positive effect on the human body has been quite well documented in the scientific literature. Although unsaturated fatty acids are fairly well known and their anti-cancer properties are investigated and described, there is literally scarce of literature data on TA. Our previous papers have shown that TA has a positive effect on healthy human fibroblasts by reducing oxidative stress level and that TA exhibits toxicity towards breast cancer cells by stimulating apoptosis through an increased level of oxidative stress [11,12]. In our preliminary study we also did an experiment regarding the mixtures of TA with selected herbicides frequently used in Poland and in EU on three breast cancer cell lines and one normal healthy cell line obtained from mammary gland [13]. Based on conducted experiments we concluded that TA in a dose–dependent manner may exert some toxicological effects in analyzed cells subjected also to herbicides. Therefore, as a next step, in this study we want to start investigating the mechanisms by which these two compounds may interact with each other and therefore influence growth and development of breast cancer cells.

First, the influence of analyzed compounds on the ZR-75-1 cell line proliferation was investigated. The MTT test was the basic experiment on the basis of which the concentrations were selected for further determinations. Cells under analysis were subjected to wide range of TA and Mes concentrations for 24 h and 48 h. The relative cell viability was monitored, and subsequently, one of Mes concentrations (0.05 µM) was selected for the experiment with the combination of two tested compounds. In the third part of the experiment, cells were pretreated with TA in the wide range of concentrations, and then Mes in the concentration of 0.05 µM was added. The most significant declines in Mes-treated cells viability were noticed as a result of 100 µM and 200 µM of TA treatment. The presented results are in agreement with our previously published data regarding TA influence on MCF-7 cells, where we indicated a decline in cancer cell proliferation and viability caused by TA [12]. Literature data has indicated that pesticides may stimulate cancer cells proliferation through different mechanisms, e.g., glyphosate stimulates human breast cancer cells growth through estrogen receptors pathways, diuron acts in a tissue-specific manner and ROS play a role in its toxicity, and bifenox and dichlobenil exhibit enhancing effects on oxidative stress, simultaneously stimulating cancer cell proliferation and inhibiting apoptosis [17,18,19]. There is a large amount of literature data indicating a link between elevated levels of oxidative stress and a simultaneous increase in tumor cell proliferation [20,21,22]. Therefore, in our work, we focused primarily on the analysis of various parameters of oxidative stress. Cancer cells of different types are characterized by high level of ROS as compared to normal cells. This is primarily due to genetic disorders which appear in cancer cells, resulting in uncontrolled proliferation. In order to analyze the possible inhibiting effect of TA on the proliferation of Mes-treated cells, we conducted an MTT assay. The results indicate that TA exhibits antiproliferative properties. 

In our studies, we noticed a decline in the amount of ROS in Mes-treated cells preincubated with TA, which may be associated with the cytotoxic effect of TA in cancer cells. Cancer cells usually use elevated level of oxidative stress caused, among others, by ROS generation in order to reduce the body’s antioxidant protection. This allows the initial defeat of the first defense line against metastasis and angiogenesis, which are key stages in the development and progression of cancer [23,24]. This is in agreement with our research results indicating the relationship between an observed increase in ROS content and an increase in proliferation in Mes-treated cells and the decrease in ROS content in TA preincubated cells with a decrease in their proliferation level.

Many compounds of natural origin from the cytokinin group have antioxidant activity in healthy cells and pro-oxidative in cancer cells, which we also showed in our previous papers [12,25]. TA, as an unsaturated fatty acid, shows an increased susceptibility to oxidative processes. According to the literature, breast cancer cells are also definitely predisposed to oxidation of the macromolecules, which build them as compared to healthy cells. Unsaturated fatty acids have been shown to induce increased synthesis in *inter alia* lipid hydroperoxides in lipids that are part of cell membranes [26,27]. According to O’Shea M. et al., conjugated linoleic acid causes an increase in lipid peroxidation in breast cancer cells with a simultaneous decrease in cell proliferation [28]. We observed similar results in our research. We noticed increases in TBARS levels, which are correlated with changes in the content of SH groups. Incubation with TA for 48 h caused a decrease in thiol groups, even in Mes-treated cells. Mes stimulates the growth of cancer cells and causes an increase in the level of SH groups, which can probably also increase their resistance to oxidative stress and possible damage and enhance proliferation. Changes in the level of TBARS content and SH groups were also accompanied by changes in the GSH/GSSG ratio, particularly decreases under the influence of preincubation with 200 µM TA in cells treated with Mes. The correspondingly high level of GSH, which is one of the most important low molecular weight antioxidants in breast cancer cells, is usually correlated with the resistance of these cells to the induction of apoptosis and with their increased proliferation [29]. Our results indicate statistically significant decreases in GSH level under the influence of 200 µM TA in cells treated with Mes, with simultaneous statistically significant increases in the level of effector caspases 3/7 activity. The analysis of the activity of caspases 3/7, confirmed by the results obtained from flow cytometry and fluorescence microscopy, demonstrates that, at a concentration of 0.05 µM, Mes induces a decrease in the percentage of apoptotic cells as compared to control and to TA-treated cells. After both 24 h and after 48 h, we observed that TA at 200 µM significantly induced apoptosis even in combination with Mes, which may mean that TA is capable of overcome activity of pesticide and stimulate apoptosis in breast cancer cells. 

Due to the application of TA, especially at a concentration of 200 µM, we noticed a significant increase in the activity of caspase 3/7, a decrease in ROS content, and a decrease in GSH content. However, it should be noted that the cells antioxidant defense system are divided into two parts: Enzymatic and nonenzymatic. Primary endogenous antioxidants are superoxide dismutase (SOD), catalase and glutathione peroxidase [30]. Our results clearly show that TA caused an increase in oxidative toxicity in Mes-treated ZR-75-1 cells. It was manifested by a decrease in GPx activity and GSH/GSSG ratio and increase in TBARS content. Lipid hydroperoxides and other ROS are a major cause of oxidative damage within the cell membrane lipids, leading to increase in TBARS content. The GSH pool in the cells decreases due to an excessive generation of lipid peroxidation products and other oxygen species [31]. In turn, an excess in TA-induced ROS generation causes a significant decline in GSH synthesis and inhibition of antioxidant enzymes [32]. The reduced form of glutathione is an antioxidant low in molecular mass for appropriate cell integrity and redox balance, although GPx is an antioxidant enzyme, which contains selenium and its main role is scavenging of ROS. Catalase in breast cancer cells is characterized by high activity and expression level [33]. In our research, a decrease in catalase activity was observed under the influence of Mes and both analyzed concentrations of TA. However, under the influence of TA mixed with Mes, significant increases were noticed in both analyzed TA concentrations, especially after 48 h incubation. The presented results are consistent with literature data, indicating that catalase activity is induced in MCF-7 breast cancer cell line exposed to conjugated linoleic acid [34]. However, SOD activity, similar to GPx activity under the influence of Mes in TA-preincubated cells, was significantly lower as compared to the control or to the TA-treated and Mes-treated cells. Mes enhanced the activity of both SOD and GPx, but TA was effective enough to withstand the stimulating effect of the Mes and reduce the activity of the enzymes studied. Ding WQ et al. also observed that cancer cell treatment with docosahexaenoic acid reduced significantly SOD1 expression [35]. Literature data describing in vivo studies have shown divergent results of analyses regarding the effect of fatty acids on antioxidant enzyme activity. Some studies have indicated higher activity of antioxidant enzymes analyzed in animals consuming PUFA-enriched feed, while others have indicated that PUFAs caused a decline in the activity of these enzymes in the tissues of noncancerous rats [36,37]. In the present study, we found that TA inhibits SOD activity in Mes-treated ZR-75-1 cells, which has not been reported previously. Selected unsaturated fatty acids are known to modulate genes expression in malignant cells [38]. After being transported into cell nucleus, fatty acids such as TA are bounded to peroxisome proliferator-activated receptor [39]. This receptor response element is in the SOD1 gene promoter in rats [40]. Therefore, the transcription of the SOD1 gene could be influenced by TA in ZR-75-1 cells. On the other hand, SOD1 mRNA destabilization could be influenced by DHA, which subsequently causes its lower expression. The target reduction of SOD activity was a way to increase intracellular peroxide amount, hence causing an increase in mitochondrial damage and stimulating apoptosis in cancer cells [41].

In our experiments, we observed a 45% decrease of SOD activity in the ZR-75-1 cells treated with 200 µM TA and incubated with Mes. According to our biplot analysis results, this was not correlated the growth rate of cells but was significantly correlated with the effect on TA-induced lipid peroxidation and ROS content after 24 h and with GPx activity, GSH/GSSG ratio, and SH group content. Our results support the idea that compounds, which influence antioxidant enzymes activity and oxidative balance in cancer cells, could be applied in the elimination of tumor cells through the induction of apoptosis.

## 5. Conclusions

The presented results allow us to conclude that TA may act as pro-oxidative and pro-apoptotic agent against Mes-stimulated breast cancer growth and development. TA could be considered as a plant, alternative source of unsaturated fatty acids that can eliminate the positive effect of pesticides on the growth and development of breast cancer cells. By stimulating oxidative stress and inhibiting the enzymatic antioxidative defense system in cancer cells, this compound can inhibit the growth and development of breast cancer. It should be also mentioned that TA acts as a pro-oxidative and pro-apoptotic agent in other breast cancer cell lines, simultaneously acting as antioxidant in normal human cells. Due to its unique properties, it could be considered as an important food ingredient. Exposure to herbicides present in food is dangerous for both healthy people and certainly for women diagnosed with breast cancer. This is also evidenced by the results of our research showing the positive and stimulating effect of Mes on the development and growth of cancer cells. However, TA seems to be a compound with high anti-cancer potential, which may endure the negative impact of herbicides on the human body.

## References

[1] Jabłońska-Trypuć A., Wołejko E., Wydro U., Butarewicz A. (2017). The impact of pesticides on oxidative stress level in human organism and their activity as an endocrine disruptor. J. Environ. Sci. Health B.

[2] Hamilton D., Crossley S. (2004). Pesticide Residues in Food and Drinking Water: Human Exposure and Risks.

[3] Mitchell G., Bartlett D.W., Fraser T.E.M., Hawkes T.R., Holt D.C., Townson J.K., Wichert R.A. (2001). Mesotrione: A new selective herbicide for use in maize. Pest Manag. Sci..

[4] Chrétien F., Giroux I., Thériault G., Patrick G., Corriveau J. (2017). Surface Runoff and Subsurface Tile Drain Losses of Neonicotinoids and Companion Herbicides at Edge-Of-Field. Environ. Pollut..

[5] Norris S.R., Barrette T.R., Della Penna D. (1995). Genetic dissection of carotenoid synthesis in arabidopsis defines plastoquinone as an essential component of phytoene desaturation. Plant Cell.

[6] Zhang F., Yao X., Sun S., Wang L., Liu W., Jiang X., Wang J. (2019). Effects of mesotrione on oxidative stress, subcellular structure, and membrane integrity in Chlorella vulgaris. Chemosphere.

[7] Bonnet J.L., Bonnemoy F., Dusser M., Bohatier J. (2008). Toxicity assessment of the herbicides sulcotrione and mesotrione toward two reference environmental microorganisms: Tetrahymena pyriformis and Vibrio fischeri. Arch. Environ. Contam. Toxicol..

[8] Bonefeld-Jørgensen E.C., Andersen H.R., Rasmussen T.H., Vinggaard A.M. (2001). Effect of highly bioaccumulated polychlorinated biphenyl congeners on estrogen and androgen receptor activity. Toxicology.

[9] Ellsworth R.E., Mamula K.A., Costantino N.S., Deyarmin B., Kostyniak P.J., Chi L.H., Shriver C.D., Ellsworth D.L. (2015). Abundance and distribution of polychlorinated biphenyls (PCBs) in breast tissue. Environ. Res..

[10] Engel L.S., Werder E., Satagopan J., Blair A., Hoppin J.A., Koutros S., Lerro C.C., Sandler D.P., Alavanja M.C., Beane Freeman L.E. (2017). Insecticide Use and Breast Cancer Risk among Farmers’ Wives in the Agricultural Health Study. Environ. Health Perspect..

[11] Jabłońska-Trypuć A., Pankiewicz W., Czerpak R. (2016). Traumatic Acid Reduces Oxidative Stress and Enhances Collagen Biosynthesis in Cultured Human Skin Fibroblasts. Lipids.

[12] Jabłońska-Trypuć A., Krętowski R., Wołejko E., Wydro U., Butarewicz A. (2019). Traumatic acid toxicity mechanisms in human breast cancer MCF-7 cells. Regul. Toxicol. Pharmacol..

[13] Jabłońska-Trypuć A., Wydro U., Wołejko E., Butarewicz A. (2019). Toxicological Effects of Traumatic Acid and Selected Herbicides on Human Breast Cancer Cells: In Vitro Cytotoxicity Assessment of Analyzed Compounds. Molecules.

[14] Carmichael J., DeGraff W.G., Gazdar A.F., Minna J.D., Mitchell J.B. (1987). Evaluation of a tetrazolium-based semiautomated colorimetric assay: Assessment of chemosensitivity testing. Cancer Res..

[15] Jabłońska-Trypuć A., Krętowski R., Kalinowska M., Świderski G., Cechowska-Pasko M., Lewandowski W. (2018). Possible Mechanisms of the Prevention of Doxorubicin Toxicity by Cichoric Acid—Antioxidant Nutrient. Nutrients.

[16] Krętowski R., Kusaczuk M., Naumowicz M., Kotyńska J., Szynaka B., Cechowska-Pasko M. (2017). The Effects of Silica Nanoparticles on Apoptosis and Autophagy of Glioblastoma Cell Lines. Nanomaterials.

[17] Thongprakaisang S., Thiantanawat A., Rangkadilok N., Suriyo T., Satayavivad J. (2013). Glyphosate induces human breast cancer cells growth via estrogen receptors. Food Chem. Toxicol..

[18] Huovinen M., Loikkanen J., Naarala J., Vähäkangas K. (2015). Toxicity of diuron in human cancer cells. Toxicol. Vitro.

[19] Jabłońska-Trypuć A., Wydro U., Serra-Majem L., Wołejko E., Butarewicz A. (2019). The Analysis of Bifenox and Dichlobenil Toxicity in Selected Microorganisms and Human Cancer Cells. Int. J. Environ. Res. Public Health.

[20] Kumari S., Badana A.K., Malla R. (2018). Reactive Oxygen Species: A Key Constituent in Cancer Survival. Biomark. Insights.

[21] Coughlin S.S. (2018). Oxidative Stress, Antioxidants, Physical Activity, and the Prevention of Breast Cancer Initiation and Progression. J. Environ. Health Sci..

[22] Jezierska-Drutel A., Rosenzweig S.A., Neumann C.A. (2013). Role of oxidative stress and the microenvironment in breast cancer development and progression. Adv. Cancer Res..

[23] Hecht F., Pessoa C.F., Gentile L.B., Rosenthal D., Carvalho D.P., Fortunato R.S. (2016). The role of oxidative stress on breast cancer development and therapy. Tumor Biol..

[24] Gorrini C., Harris I.S., Mak T.W. (2013). Modulation of oxidative stress as an anticancer strategy. Nat. Rev. Drug Discov..

[25] Jabłońska-Trypuć A., Matejczyk M., Czerpak R. (2016). N6-benzyladenine and kinetin influence antioxidative stress parameters in human skin fibroblasts. Mol. Cell. Biochem..

[26] Takeda S., Horrobin D.F., Manku M., Sim P.G., Ells G., Simmons V. (1992). Lipid peroxidation in human breast cancer cells in response to gamma-linolenic acid and iron. Anticancer Res..

[27] Menendez J.A., Ropero S., Mehmi I., Atlas E., Colomer R., Lupu R. (2004). Overexpression and hyperactivity of breast cancer-associated fatty acid synthase (oncogenic antigen-519) is insensitive to normal arachidonic fatty acid-induced suppression in lipogenic tissues but it is selectively inhibited by tumoricidal alpha-linolenic and gamma-linolenic fatty acids: A novel mechanism by which dietary fat can alter mammary tumorigenesis. Int. J. Oncol..

[28] O’Shea M., Devery R., Lawless F., Murphy J., Stanton C. (2000). Milk fat conjugated linoleic acid (CLA) inhibits growth of human mammary MCF-7 cancer cells. Anticancer Res..

[29] Malla J.A., Umesh R.M., Yousf S., Mane S., Sharma S., Lahiri M., Talukdar P. (2020). A Glutathione Activatable Ion Channel Induces Apoptosis in Cancer Cells by Depleting Intracellular Glutathione Levels. Angew. Chem. Int. Ed. Engl..

[30] Chio I.I.C., Tuveson D.A. (2017). ROS in cancer: The burning question. Trends Mol. Med..

[31] Nazıroglu M. (2007). New molecular mechanisms on the activation of TRPM2 channels by oxidative stress and ADP-ribose. Neurochem. Res..

[32] Nazıroglu M., Tokat S., Demirci S. (2012). Role of melatonin on electro-magnetic radiation-induced oxidative stress and Ca2þ signaling molecular pathways in breast cancer. J. Recept. Signal Transduct. Res..

[33] Kattan Z., Minig V., Leroy P., Dauça M., Becuwe P. (2008). Role of manganese superoxide dismutase on growth and invasive properties of human estrogen-independent breast cancer cells. Breast Cancer Res. Treat..

[34] O’Shea M., Stanton C., Devery R. (1999). Antioxidant enzyme defence responses of human MCF-7 and SW480 cancer cells to conjugated linoleic acid. Anticancer Res..

[35] Ding W.Q., Vaught J.L., Yamauchi H., Lind S.E. (2004). Differential sensitivity of cancer cells to docosahexaenoic acid-induced cytotoxicity: The potential importance of down-regulation of superoxide dismutase 1 expression. Mol. Cancer Ther..

[36] Venkatraman J.T., Chandrasekar B., Kim J.D., Fernandes G. (1994). Effects of n-3 and n-6 fatty acids on the activities and expression of hepatic antioxidant enzymes in autoimmune-prone NZB × NZW F1 mice. Lipids.

[37] Venkatraman J.T., Angkeow P., Satsangi N., Fernandes G. (1998). Effects of dietary n-6 and n-3 lipids on antioxidant defense system in livers of exercised rats. J. Am. Coll. Nutr..

[38] Hughes-Fulford M., Chen Y., Tjandrawinata R.R. (2001). Fatty acid regulates gene expression and growth of human prostate cancer PC-3 cells. Carcinogenesis.

[39] Huang H., Starodub O., McIntosh A., Kier A.B., Schroeder F. (2002). Liver fatty acid-binding protein targets fatty acids to the nucleus. Real time confocal and multiphoton fluorescence imaging in living cells. J. Biol. Chem..

[40] Chang M.S., Yoo H.Y., Rho H.M. (1999). Positive and negative regulatory elements in the upstream region of the rat Cu/Zn-superoxide dismutase gene. Biochem. J..

[41] Huang P., Feng L., Oldham E.A., Keating M.J., Plunkett W. (2000). Superoxide dismutase as a target for the selective killing of cancer cells. Nature.

